# Potential Role of mRNAs and LncRNAs in Chronic Intermittent Hypoxia Exposure-Aggravated Atherosclerosis

**DOI:** 10.3389/fgene.2020.00290

**Published:** 2020-04-09

**Authors:** Jing Zhang, Chaowei Hu, Xiaolu Jiao, Yunyun Yang, Juan Li, Huahui Yu, Yanwen Qin, Yongxiang Wei

**Affiliations:** ^1^Beijing Anzhen Hospital, Capital Medical University, Beijing, China; ^2^Key Laboratory of Remodeling-Related Cardiovascular Diseases, Ministry of Education, Beijing, China; ^3^Key Laboratory of Upper Airway Dysfunction-Related Cardiovascular Diseases, Beijing, China; ^4^Beijing Institute of Heart Lung and Blood Vessel Disease, Beijing, China

**Keywords:** atherosclerosis, obstructive sleep apnea, chronic intermittent hypoxia, microarray, long non-coding RNAs

## Abstract

Atherosclerosis is the pathological basis of cardiovascular disease. Obstructive sleep apnea (OSA) aggravates atherosclerosis, and chronic intermittent hypoxia (CIH) as a prominent feature of OSA plays an important role during the process of atherosclerosis. The mechanisms of CIH in the development of atherosclerosis remain unclear. In the current study, we used microarray to investigate differentially expressed mRNAs and long non-coding RNAs (lncRNAs) in aorta from five groups of ApoE^–/–^ mice fed with a high-fat diet and exposed to various conditions: normoxia for 8 weeks, CIH for 8 weeks, normoxia for 12 weeks, CIH for 12 weeks, or CIH for 8 weeks followed by normoxia for 4 weeks. Selected transcripts were validated in aorta tissues and RT-qPCR analysis showed correlation with the microarray data. Gene Ontology analysis and pathway enrichment analysis were performed to explore the mRNA function. Bioinformatic analysis indicated that short-term CIH induced up-regulated mRNAs involved in inflammatory response. Pathway enrichment analysis of lncRNA co-localized mRNAs and lncRNA co-expressed mRNAs were performed to explore lncRNA functions. The up-regulated mRNAs, lncRNA co-localized mRNAs and lncRNA co-expressed mRNAs were significantly associated with protein processing in endoplasmic reticulum pathway in atherosclerotic vascular tissue with long-term CIH exposure, suggesting that differentially expressed mRNAs and lncRNAs play important roles in this pathway. Moreover, a mRNA-lncRNA co-expression network with 380 lncRNAs, 508 mRNAs and 3238 relationships was constructed based on the correlation analysis between the differentially expressed mRNAs and lncRNAs. In summary, our study provided a systematic perspective on the potential function of mRNAs and lncRNAs in CIH-aggravated atherosclerosis, and may provide novel molecular candidates for future investigation on atherosclerosis exposed to CIH.

## Introduction

Atherosclerotic cardiovascular disease is the leading cause of death worldwide (Heron et al., 2009). Atherosclerosis is the pathological basis of peripheral vascular, coronary, and cerebrovascular disease (Tabas et al., 2015). Numerous traditional risk factors for atherosclerosis have been reported, including genetic predisposition, aging, hypertension, diabetes, obesity and smoking, and exposure to these risk factors plays important roles in the development of atherosclerosis. Recently, a number of novel atherosclerosis risk factors have been identified (Fruchart et al., 2004). These novel risk factors are expected to provide optimal diagnostic and therapeutic strategies for reducing cardiovascular and cerebrovascular events in patients.

Obstructive sleep apnea (OSA), characterized by recurrent pharyngeal collapses during sleep (Levy et al., 2015), increases linearly with age and is considered a major public health issue affecting 3–7% of the middle-aged population (30–70 years) (Duran et al., 2001; Young et al., 2002). Several studies have shown that OSA is an independent risk factor for cardiovascular disease (Peker et al., 2002; Bradley and Floras, 2009; Ma et al., 2016), resulting in hypertension (Lavie et al., 2000), stroke (Yaggi et al., 2005), cardiac events (Shah et al., 2010), heart failure (Khattak et al., 2018), arrhythmia (Rossi et al., 2013), and sudden death in the night (Gami et al., 2013). Many clinical and research studies have provided evidence for a correlation between OSA and atherosclerosis (Song et al., 2015) and demonstrated that OSA changes the progression and outcomes of atherosclerosis. OSA is associated with increased carotid intima-media thickness (IMT) and plaque occurrence (early atherosclerotic lesions), independent of cardiovascular risk factors or cardiovascular diseases (Baguet et al., 2005). OSA is also independently associated with arterial stiffness (Drager et al., 2007b, 2010), which is a strong predictor of late cardiovascular event. Although evidence supporting the aggravation of atherosclerosis by OSA is relatively strong, relatively little is known about the underlying molecular mechanisms linking OSA to atherosclerosis.

A prominent feature of OSA pathophysiology is chronic intermittent hypoxia (CIH), which is long-term exposure to repeated episodes of reoxygenation following hypoxia induced by airflow obstruction during sleep (Neubauer, 2001; Dempsey et al., 2010). CIH is a major underlying culprit for OSA-induced cardiovascular and cerebrovascular complications (Kohler and Stradling, 2010; Ma et al., 2016). CIH promotes atherosclerotic plaque formation in ApoE-deficient mice fed a normal chow diet (Fang et al., 2012) or high cholesterol diet (Jun et al., 2010). In addition, continuous positive airway pressure (CPAP) therapy for OSA patients ameliorates atherosclerosis by reducing IMT and carotid to femoral artery pulse wave velocity (CF-PWV) (Drager et al., 2007a). The core pathological manifestations of OSA depend on the strong correlation between CIH and atherosclerosis, and CIH accelerates the development of atherosclerosis. The roles and mechanisms of CIH in the development of atherosclerosis are still unknown.

Long non-coding RNAs (lncRNAs) are non-protein-coding RNAs > 200 nucleotides in length characterized by RNA polymerase II processing, a 5′ cap, and 3′ polyadenylation (Jian et al., 2016; Liu et al., 2017). LncRNAs do not encode proteins but regulate gene expression through epigenetic, transcriptional and post-transcriptional regulation mechanisms (Jian et al., 2016). LncRNAs can fold into secondary or higher order structures to more flexibly target proteins or gene sites (Guttman and Rinn, 2012). LncRNAs exert critical roles on regulating biological processes such as cell survival, cell cycle, and metabolism (Hung et al., 2011; Grammatikakis et al., 2014). Endothelial cells, vascular smooth muscle cells and macrophages are the primary cells leading to atherosclerotic lesion formation (Tabas et al., 2015). Previous studies have demonstrated that lncRNAs regulate the functions of endothelial cells, smooth muscle cells and macrophages, suggesting that lncRNAs are emerging important players in the progression of atherosclerosis (Jian et al., 2016; Zhou et al., 2016). However, the underlying mechanisms of lncRNAs in CIH aggravating atherosclerosis are poorly understood, and the study of lncRNAs in CIH aggravating atherosclerosis is scarce.

In the present study, we analyzed lncRNAs and mRNAs by microarray at different time points during the atherosclerosis process in ApoE-deficient mice fed a high-fat diet under CIH or normoxia conditions. We performed a systematic analysis of the functionality of lncRNAs and mRNAs during the progression of atherosclerosis under CIH exposure. These findings will shed new light on the roles of OSA and CIH on atherosclerosis.

## Materials and Methods

### Animals

Male 8-week-old ApoE^–/–^ mice were purchased from Beijing Hua Fukang Biotechnology, Co. Ltd. (Beijing, China). The ApoE^–/–^ mice were randomly divided into five groups: Nor 8W (mice in normoxia for 8 weeks), CIH 8W (mice in CIH for 8 weeks), Nor 12W (mice in normoxia for 12 weeks), CIH 12W (mice in CIH for 12 weeks), and CIH 8W + Nor 4W groups (mice in CIH for 8 weeks followed by normoxia for 4 weeks). All ApoE^–/–^ mice were fed with high-fat feed (21% fat and 0.15% cholesterol), which was purchased from Beijing Hua Fukang Biotechnology, Co. Ltd. The aorta from the five groups (5 in Nor 8W, 5 in CIH8W, 5 in Nor 12W, 4 in CIH 12W, 4 in CIH 8W + Nor 4W) was used for microarray analysis. All animal procedures conformed to the Guide for the Care and Use of Laboratory Animals published by the US National Institutes of Health (NIH publication no. 85-23, revised 1996). This study was approved by the Institutional Animal Care and Use Committee of Capital Medical University, Beijing, China.

### Intermittent Hypoxia

ApoE^–/–^ mice were placed into a plexiglass chamber connected to OxyCycler A84 (BioSpherix, Redfield, NY, United States). During each period of intermittent hypoxia, the fractional inspired O_2_ (FiO_2_) was reduced from 20.9 to 5.0–6.0% over 120 s and re-oxygenated to 20.9% in the subsequent 60 s period. This cycle was repeated every 180 s from 09:00 to 21:00 daily to coincide with mouse sleep cycles. Normoxia was delivered at identical flow rates with a constant FiO_2_ of 20.9%.

### Atherosclerotic Lesion Analysis

The day following the last exposure period, ApoE^–/–^ mice were intraperitoneally administered 1% pentobarbital sodium anesthesia (5 mg/kg). Mice were sacrificed and transcardially perfused with PBS. For analysis of atherosclerotic lesions on aorta, the aortic tree was opened longitudinally starting from the aortic root to the iliac bifurcation, pinned, fixed in 10% buffered formalin, and stained with Oil Red O. The atherosclerotic burden was quantified in a blind fashion using NIS-Elements F Ver4.60.00 software (Nikon, Tokyo, Japan) and expressed as the ratio of Oil Red O-positive lesions to the total aortic area.

### Total RNA Extraction

Total RNA was extracted from mouse aorta using TRIZOL reagent (Life Technologies, Foster City, CA, United States) following the manufacturer’s instructions, and digested with DNase I at 37°C for 15 min to remove any contaminating DNA. The RNA was further purified with the RNeasy Kit (Qiagen, Hilden, Germany). The RNA quantity and quality were measured by a NanoDrop 1000 spectrophotometer (Thermo Fisher Scientific, Waltham, MA, United States), and the RNA integrity was assessed by standard denaturing agarose gel electrophoresis.

### Microarray Analysis

The transcriptome was analyzed using the Clariom^TM^ D Array (Mouse; Affymetrix, Thermo Fisher Scientific), which contains transcripts of 22413 coding RNAs and 43543 non-coding RNAs. The RNA labeling and microarray hybridization were carried out according to the GeneChip WT PLUS Reagent Kit User Guide (P/N 703174 Rev.5, Affymetrix, Inc., Santa Clara, CA, United States). Briefly, the total RNA was amplified and transcribed into cRNA along the entire length of the transcripts without 3′ bias. The sense-strand cDNA was synthesized by reverse transcription of cRNA. The sense-strand cDNA was fragmented and labeled with biotin. The sense-strand cDNA was hybridized to the Clariom^TM^ D Array at 45°C for 16 h according to the manufacturer’s instructions. After hybridization, the microarrays were washed and then stained in Affymetrix Fluidics Station 450 (Affymetrix) and scanned using theGene-Chip^®^ scanner 3000 7G (Affymetrix) following the manufacturer’s instructions. Affymetrix^®^ GeneChip Command Console (AGCC) was used to analyze acquired array images. Raw signal intensities were normalized using Transcriptome Analysis Console (TAC) 4.0 software (Applied Biosystems, Thermo Fisher Scientific). Microarray data were deposited in NCBI with the GEO accession code GSE145221.

### Quantitative Real-Time PCR

Microarray data were validated by qRT-PCR. In brief, total RNA was extracted by TRIZOL reagent (Life Technologies, Thermo Fisher Scientific) and purified with the RNeasy Kit (Qiagen) according to the manufacturer’s instructions. M-MLV reverse transcription was used to synthesize cDNA (Promega, Madison, WI, United States). Quantitative real-time PCR was carried out with SYBR Green PCR mix (Applied Biosystems) on the ABI 7900HT qPCR system (Applied Biosystems). Four mRNAs and two lncRNAs were randomly selected for validation. The following primers were used: Cox8b (5′-TGTGGGGATCTCAGCCATAGT-3′ forward; 5′-AGTGGGCTAAGACCCATCCTG-3′ reverse), Hsp90b1 (5′-GTTCGTCAGAGCTGATGATGAA-3′ forward; 5′-GCGTTTAACCCATCCAACTGAAT-3′ reverse), Ucp1 (5′-GTGAACCCGACAACTTCCGAA-3′ forward; 5′-TGCCAGG CAAGCTGAAACTC-3′ reverse), Nr1d2 (5′-CAGGAGGTG TGATTGCCTACA-3′ forward; 5′-GGACGAGGACTGGAAGC TATT-3′ reverse), Gm13910 (5′-ATCCGTCCTTCCTC ACTGGA-3′ forward; 5′-TGATTAGCATCGCAGAAGCG-3′ reverse), Gm5421 (5′-CGAGGACATGGGACGATCAG-3′ forward; 5′-AACGATTCCACCCACATCCC-3′ reverse). RPS18 (Cat. Number B661301, Sango Biotech, Shanghai, China) was used as the internal control. The relative expression was calculated using the 2^–Δ^ ^Δ^ ^CT^ method and RPS18 was used to normalize the quantitative real-time data. Results were expressed as relative fold changes compared with levels in Nor 8W. After normalized microarray data as relative fold changes compared with levels in Nor 8W, Pearson’s correlation coefficient was further calculated for each gene to quantify the consistency between microarray data and qRT-PCR data.

### Bioinformatics Data Analysis

After normalization of the raw data, we used the limma R package (version: 3.36.5) to filter the differentially expressed genes. Limma R used moderated F-statistic to filter the multi-group differentially expressed genes. Empirical Bayes moderation was used to correct the *p*-values (Pan et al., 2018). The threshold set for up- and down-regulated genes was a fold change > 1.1 and *p* < 0.05. Gene Ontology (GO) and Kyoto Encyclopedia of Genes and Genomes (KEGG) pathway analysis were used to investigate the roles of the differentially expressed mRNAs. The roles of the differentially expressed lncRNAs were investigated by KEGG pathway annotations of co-localized mRNAs and co-expressed mRNAs. The neighboring (20 kb upstream or downstream) protein-coding genes of the differentially expressed lncRNAs were selected as co-localized mRNAs. For GO analysis^1^, the corresponding genes were divided into three aspects by enrichment analysis, including biological process (BP), molecular function (MF) and cellular component (CC). KEGG pathway analysis was performed to examine the significant pathways of the differentially expressed genes^2^. The GO and KEGG pathway analysis were performed using R software with ggplot2 package. The mRNA-lncRNA co-expression network analysis was performed to assess functional annotation. The networks were built based on positive or negative correlations according to the normalized signal intensity of individual transcripts. The mRNAs and lncRNAs with significant differential expression between the five treatment groups were selected for the network analysis. The Pearson’s correlation coefficient value was calculated for mRNA-lncRNA pairs. The strong correlated pairs (Pearson’s correlation coefficient ≥ 0.9 and *p* < 0.05) were selected for illustrating the co-expression network. Gene co-expression network was constructed from the preprocessed files using R package “weighted correlation network analysis” (Song et al., 2012). Following the protocol for constructing gene co-expression network using multiple datasets (Stuart et al., 2003), we first calculated Pearson correlation matrix for each dataset. We then obtained an overall weighted correlation matrix based on the number of samples used in that dataset. The visualization of network was built by software Cytoscape (version: 3.6.0).

### Statistical Analysis

Data were expressed as means ± standard deviation. One-way ANOVA followed by Bonferronic *post hoc* test for comparisons between more than two groups was conducted in atherosclerotic lesion analysis. Limma R used moderated F-statistic to filter the multi-group differentially expressed genes. Empirical Bayes moderation was used to correct the *p*-values. GO analysis and pathway analysis were performed using the two side Fisher’s exact test and Benjamini–Hochberg was used for multiple tests correction. Pearson’s correlation coefficient was calculated to quantify the consistency between microarray data and qRT-PCR data. The differences were considered significant at *p* < 0.05.

## Results

### CIH Exposure Aggravates Atherosclerosis in ApoE-Deficient Mice

To observe atherosclerosis with CIH exposure, we fed ApoE-deficient mice with a high-fat diet under CIH or normoxia conditions. The atherosclerotic lesions of aorta were evaluated by Oil Red O staining (Supplementary Figure S1). After 8 weeks of a high-fat diet, there were few plaques in the aorta of ApoE-deficient mice in normoxia or CIH, and there was no significant difference in plaque area between these two groups. However, the plaque area of aorta in ApoE-deficient mice under CIH for 12 weeks was significantly increased (*p* < 0.01) compared with ApoE-deficient mice in normoxia for 12 weeks. The plaque area of aorta in ApoE-deficient mice exposed to CIH for 8 weeks followed by normoxia for 4 weeks was significantly reduced (*p* < 0.01) compared with ApoE-deficient mice under CIH for 12 weeks (Supplementary Figure S1B). These results indicate that long-term CIH exposure aggravates atherosclerosis.

### Overview of Gene Expression

**FIGURE 1:**
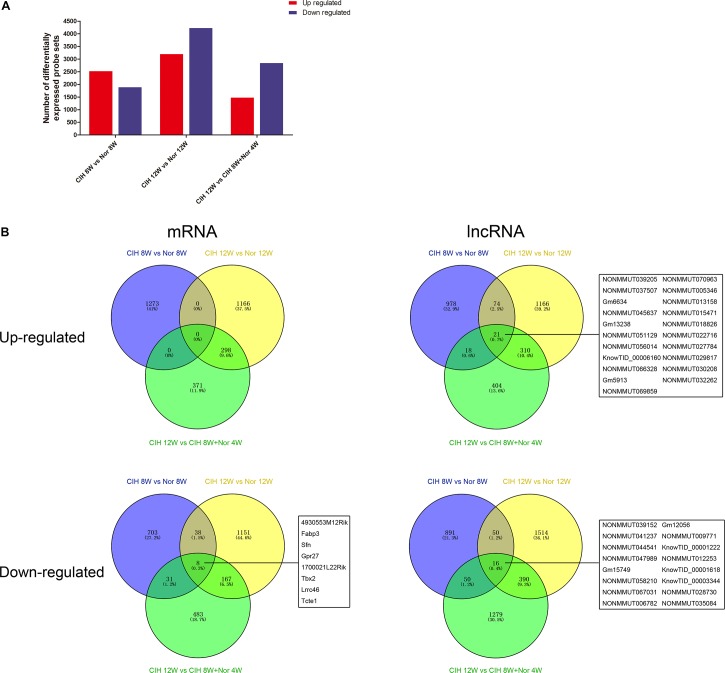
mRNA and lncRNA gene expression analysis. **(A)** Total numbers of significantly differentially up-regulated and down-regulated probe sets in the indicated groups. **(B)** Venn diagrams depicting up-regulated and down-regulated mRNAs and lncRNAs in different comparison groups. The numbers of mRNAs and lncRNAs are indicated, and the common mRNAs and lncRNAs shared among all three comparison groups are listed.

We next performed gene expression analysis between mice in CIH or normoxia for various exposure times. The number of significantly up-regulated genes was higher than the number of significantly down-regulated genes in mice exposed to CIH for 8 weeks compared with mice under normoxia for 8 weeks (Figure 1A). In contrast, the number of significantly down-regulated genes was higher than the number of up-regulated genes in mice in CIH for 12 weeks compared with mice in normoxia for 12 weeks or mice exposed to CIH for 8 weeks followed by normoxia for 4 weeks.

Analysis of significantly differentially expressed genes that were altered in the same direction in each of the three groups revealed overlapping genes. We identified 21 up-regulated lncRNAs in all three comparisons, and 8 down-regulated mRNAs and 16 down-regulated lncRNAs (Figure 1B). The top 10 changed mRNAs are listed in Table 1. The information of all expressed mRNAs and lncRNAs is presented in Supplementary Table S1. Detailed information of differentially expressed mRNAs is presented in Supplementary Table S2, and detailed information of differentially expressed lncRNAs is presented in Supplementary Table S3.

**TABLE 1:** The detailed information of the top 10 up-regulated and 10 down-regulated mRNAs.

**CIH 8W vs. Nor 8W**	**gene_name**	**Fold change**	**CIH 12W vs. Nor 12W**	**gene_name**	**Fold change**	**CIH 12W vs. CIH 8W + Nor 4W**	**gene_name**	**Fold change**
Up-regulated	Igkv8-30	3.160	Up-regulated	Cry1	2.282	Up-regulated	Arntl	3.434
	Igkv4-91	2.998		Fos	2.204		Npas2	2.346
	Spp1	2.717		2210407C18Rik	2.129		Cry1	2.313
	Chil3	2.692		Angptl7	2.014		Hsph1	2.028
	Ighv1-42	2.633		Gm13105	1.932		Creld2	1.919
	Ighv14-4	2.582		Bmp3	1.932		Chil3	1.828
	Ighv1-43	2.463		Hspb1	1.905		Nfil3	1.717
	Igkv17-121	2.433		Nr4a1	1.866		Ccl7	1.682
	Ighv1-31	2.429		Gm13295	1.853		Hspb1	1.647
	Bcl2a1a	2.416		Hspa1a	1.84		Ptprz1	1.647
Down-regulated	Cdr1	−1.459	Down-regulated	Npy	−3.945	Down-regulated	Tef	−1.693
	Dhcr24	−1.471		Mup1	−4.028		Bhlhe41	−2.028
	Npy1r	−1.486		Gm2083	−4.084		Igkv12-46	−2.056
	Pygm	−1.489		Mup19	−4.112		Per3	−2.173
	Lgi1	−1.514		Gm10801	−4.141		Tcap	−2.828
	Casq1	−1.516		Mup19	−4.141		Ciart	−3.317
	Gldn	−1.518		Mup12	−4.257		Ucp1	−3.681
	Sbspon	−1.549		Fabp7	−4.857		Nr1d2	−3.945
	F830001A07Rik	−1.569		Snap25	−5.134		Dbp	−5.205
	Fasn	−1.893		Dbh	−5.315		Nr1d1	−6.869

### Validation of Microarray Data by qRT-PCR

**FIGURE 2:**
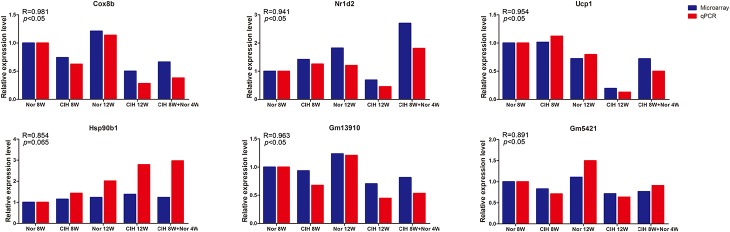
qRT-PCR validation of mRNA and lncRNA expression. Expression levels were determined by qRT-PCR analysis and the relative expression levels were calculated according to the 2^– Δ^ ^Δ^ ^Ct^ method using the RPS18 gene as an internal reference gene. qRT-PCR results are expressed as relative fold changes compared with Nor 8W. Microarray expression is also shown as relative fold changes compared with Nor 8W. R = Pearson correlation coefficient.

To validate the microarray results, four differentially expressed mRNAs (Cox8b, Nr1d2, Ucp1, Hsp90b1) and two differentially expressed lncRNAs (Gm13910 and Gm5421) were selected for qRT-PCR analysis in mouse aorta (Figure 2). The expression levels detected by qRT-PCR were consistent with the microarray results, indicating that the microarray analysis was highly reliable.

### Functional Annotation of Differentially Expressed mRNAs

**FIGURE 3:**
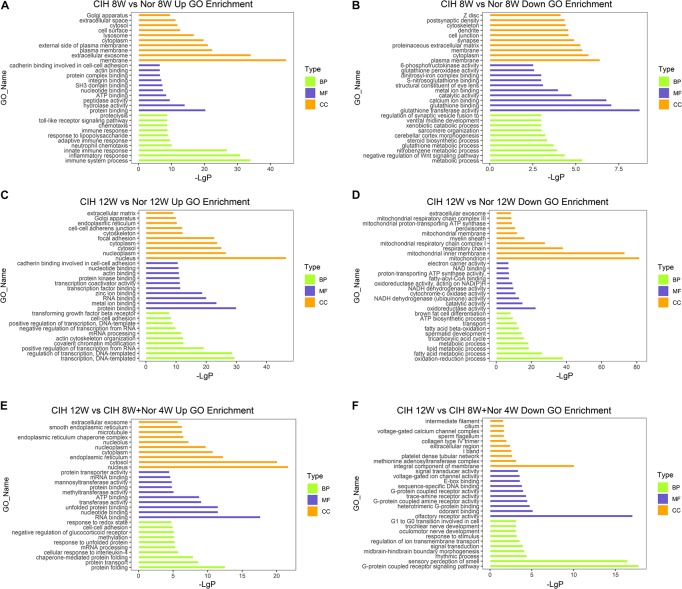
Gene ontology (GO) analysis of differentially expressed mRNAs. GO annotation of up-regulated **(A)** and down-regulated **(B)** mRNAs in the CIH 8W vs. Nor 8W comparison. GO annotation of up-regulated **(C)** and down-regulated **(D)** mRNAs in the CIH 12W vs. Nor 12W comparison. GO annotation of up-regulated **(E)** and down-regulated **(F)** mRNAs in the CIH 12W vs. CIH 8W + Nor 4W comparison. Each color represents a different aspect. Only the top 10 GO terms are listed.

To better understand the biological functions of mRNAs in atherosclerotic vascular tissue after exposure to CIH, we performed GO and KEGG pathway enrichment analysis on the mRNAs. GO analysis was performed on three different aspects including biological process (BP), cellular component (CC) and molecular function (MF) for up-regulated and down-regulated mRNAs (Figure 3 and Supplementary Table S4). The up-regulated mRNAs in the CIH 8W vs. Nor 8W comparison were genes involved in immune system process, inflammatory response and innate immune response (Figure 3A), while the down-regulated mRNAs were associated with glutathione transferase activity, glutathione binding, and glutathione peroxidase activity (Figure 3B). The up-regulated mRNAs in the CIH 12W vs. CIH 8W + Nor 4W comparison were genes involved in protein folding, protein transport and chaperone-mediated protein folding (Figure 3E).

**FIGURE 4:**
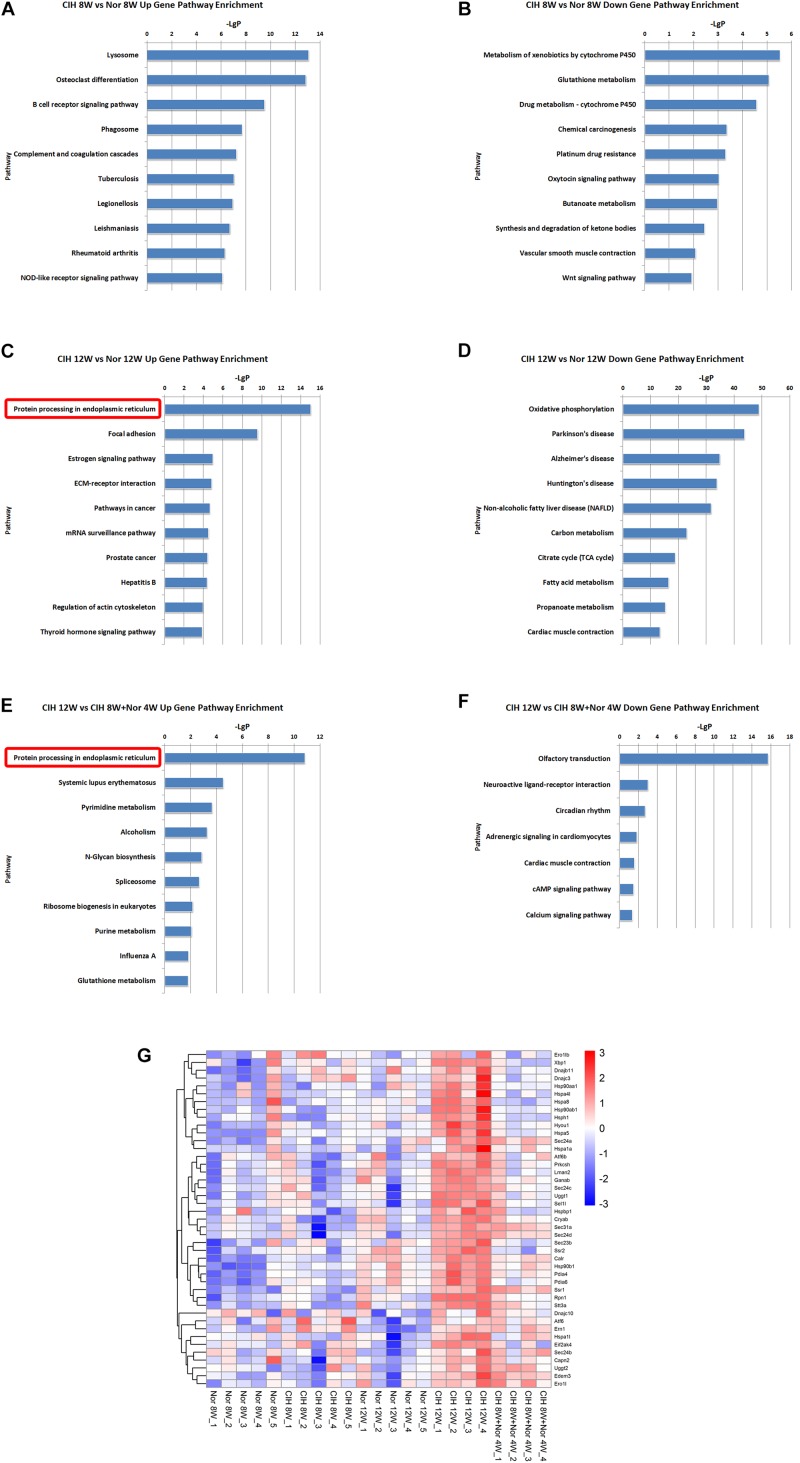
KEGG signaling pathway analysis of differentially expressed mRNA. KEGG signaling pathways of up-regulated **(A)** and down-regulated **(B)** mRNAs in the CIH 8W vs. Nor 8W comparison. KEGG signaling pathways of up-regulated **(C)** and down-regulated **(D)** mRNAs in the CIH 12W vs. Nor 12W comparison. KEGG signaling pathways of up-regulated **(E)** and down-regulated **(F)** mRNAs in the CIH 12W vs. CIH 8W + Nor 4W comparison. Only the top 10 KEGG signaling pathways are listed. **(G)** Heatmap showing the differentially expressed genes in the pathway of protein processing in endoplasmic reticulum.

KEGG pathway enrichment analysis demonstrated the possible involvement of significantly dysregulated mRNAs in related pathways and molecular interactions among genes. We identified some pathways with significant differences in mRNA expression (Supplementary Table S5). The top 10 most significant up-regulated and down-regulated pathway terms are shown in Figure 4. The protein processing in endoplasmic reticulum was the most significant pathway within the set of up-regulated mRNAs in atherosclerotic vascular tissue of ApoE-deficient mice exposed to CIH for 12 weeks (Figures 4C,E). The genes involved in the pathway of protein processing in endoplasmic reticulum were enriched and clustered for further analysis (Figure 4G).

### Functional Annotation of Differentially Expressed LncRNAs

**FIGURE 5:**
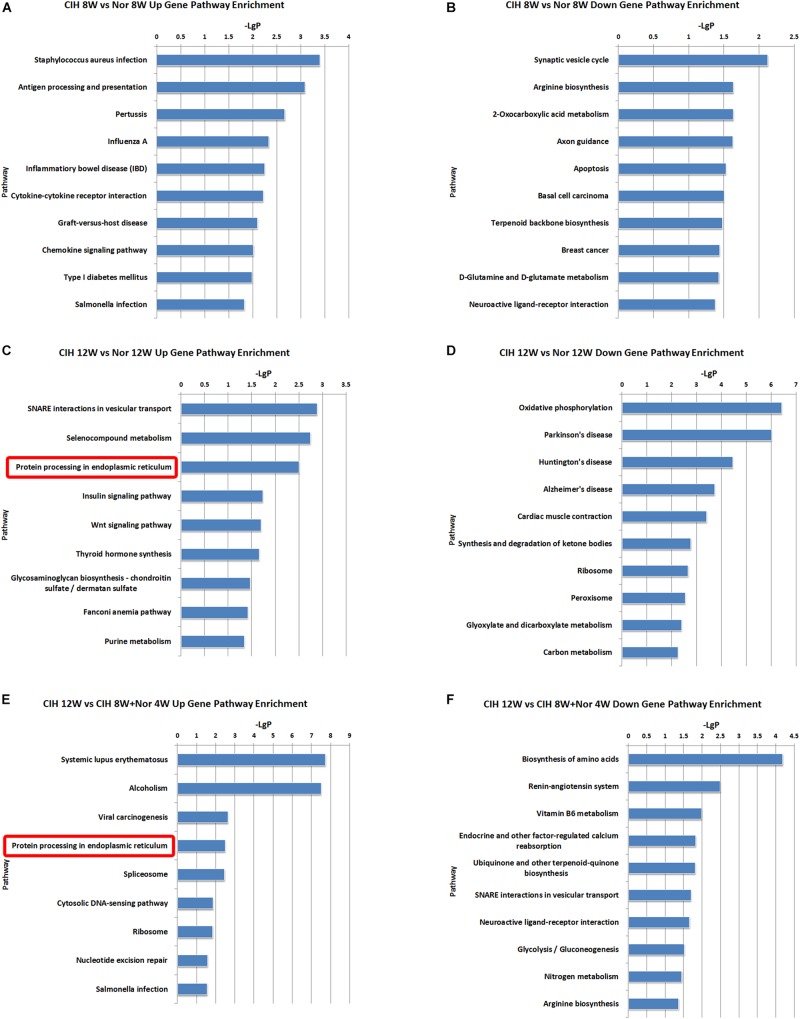
KEGG signaling pathway analysis of differentially expressed lncRNA co-localized mRNAs. KEGG signaling pathways of up-regulated **(A)** and down-regulated **(B)** lncRNA co-localized mRNAs in the CIH 8W vs. Nor 8W comparison. KEGG signaling pathways of up-regulated **(C)** and down-regulated **(D)** lncRNA co-localized mRNAs in the CIH 12W vs. Nor 12W comparison. KEGG signaling pathways of up-regulated **(E)** and down-regulated **(F)** lncRNA co-localized mRNAs in the CIH 12W vs. CIH 8W + Nor 4W comparison. Only the top 10 KEGG signaling pathways are listed.

To better understand the function of the lncRNAs identified in our dataset, we assessed two aspects of the identified lncRNAs: (1) KEGG annotation of their co-localized mRNAs; (2) KEGG annotation of their co-expressed mRNAs. The neighboring (20 kb upstream or downstream) protein-coding genes of the differentially expressed lncRNAs were selected as co-localized mRNAs. There were some pathways with significant differences in lncRNA co-localized mRNAs expression (Supplementary Table S6). The top 10 most significant up-regulated and down-regulated pathway terms are shown in Figure 5. The protein processing in endoplasmic reticulum was the overlapping pathway within the set of up-regulated lncRNA co-localized mRNAs in the CIH 12W vs. Nor 12W comparison and the CIH 12W vs. CIH 8W + Nor 4W comparison (Figures 5C,E). We compared the co-localized mRNAs of the differentially expressed lncRNAs with differentially expressed mRNAs and listed the overlapped mRNAs in Supplementary Table S7.

**FIGURE 6:**
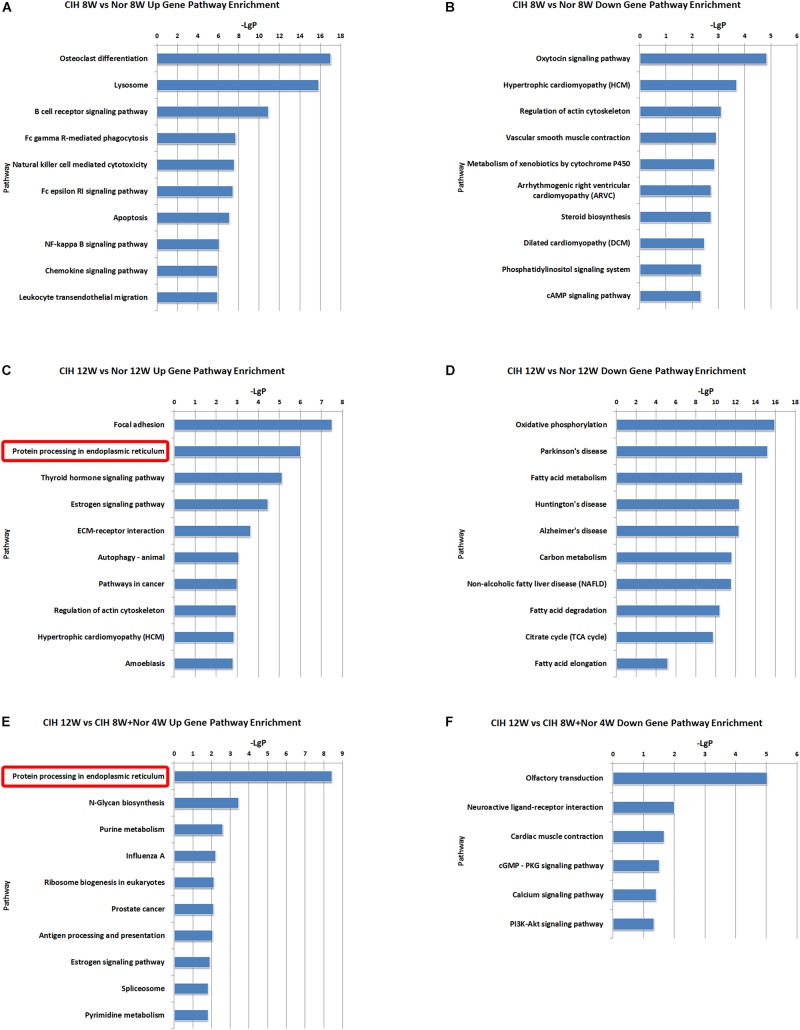
KEGG signaling pathway analysis of differentially expressed lncRNA co-expressed mRNAs. KEGG signaling pathways of up-regulated **(A)** and down-regulated **(B)** lncRNA co-expressed mRNAs in the CIH 8W vs. Nor 8W comparison. KEGG signaling pathways of up-regulated **(C)** and down-regulated **(D)** lncRNA co-expressed mRNAs in the CIH 12W vs. Nor 12W comparison. KEGG signaling pathways of up-regulated **(E)** and down-regulated **(F)** lncRNA co-expressed mRNAs in the CIH 12W vs. CIH 8W + Nor 4W comparison. Only the top 10 KEGG signaling pathways are listed.

We also identified some pathways with significant differences in lncRNA co-expressed mRNAs expression (Supplementary Table S8). The top 10 most significant up-regulated and down-regulated pathway terms were shown in Figure 6. The protein processing in endoplasmic reticulum was the overlap pathway within the set of up-regulated lncRNA co-expressed mRNAs in CIH 12W vs. Nor 12W comparison group and CIH 12W vs. CIH 8W + Nor 4W comparison group (Figures 6C,E).

We analyzed the relationship between differentially expressed mRNAs and lncRNAs, and listed mRNAs and lncRNAs with both co-localization and co-expression relationships in Table 2.

**TABLE 2:** The detailed information of mRNAs and lncRNAs with both co-localization and co-expression relationships.

	**mRNA_ name**	**mRNA fold change**	**mRNA_ chr**	**mRNA_ start**	**mRNA_ end**	**lncRNA_ name**	**lncRNA fold change**	**lncRNA_ chr**	**lncRNA_ start**	**lncRNA_ end**	**Relation**	***p*-value**	**Regulation**
CIH 8W vs. Nor 8W	Cdr1	−1.459	chrX	61183246	61185558	KnowTID_00007912	−1.184	chrX	61187019	61189437	0.98	6.601E-07	Positive
	Il2rg	1.609	chrX	101264378	101268255	NONMMUT073343	1.384	chrX	101264111	101268294	0.982	4.674E-07	Positive
	Prg4	2.14	chr1	150449412	150466165	NONMMUT003096	1.796	chr1	150453960	150456397	0.983	3.45E-07	Positive
CIH 12W vs. Nor 12W	Tubb1	−2.114	chr2	174450595	174458380	NONMMUT041650	−2.585	chr2	174458492	174459356	0.997	3.404E-09	Positive

### Construction of the mRNA-LncRNA Co-expression Network

**FIGURE 7:**
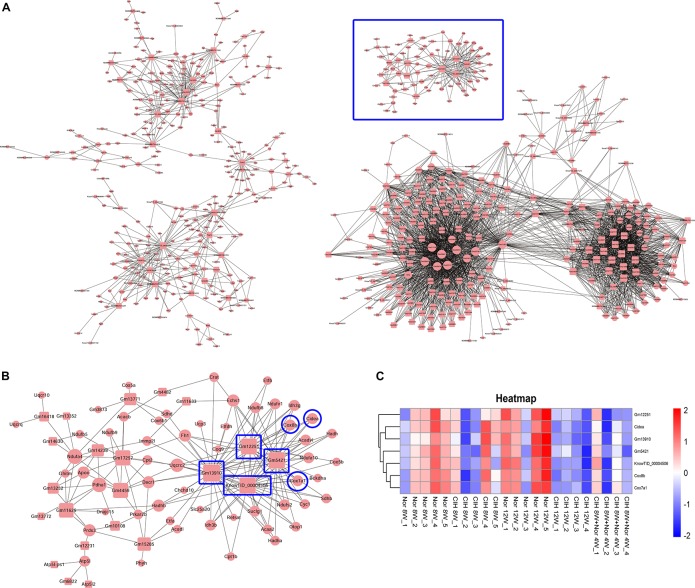
Construction of the mRNA-lncRNA co-expression network. The network represents co-expression correlations between mRNAs and lncRNAs. In the network, circles represent mRNAs and squares represent lncRNAs. Black lines indicate co-expression regulation. The size of circle represents the number of genes in the network that interact with the mRNA, and the size of square represents the number of genes in the network that interact with the lncRNA. **(A)** A total 380 lncRNAs and 508 mRNAs containing 3238 relationships were selected to generate a total network map. **(B)** One sub-network from the blue frame in **(A)** is presented in detail. **(C)** Heatmap showing the differentially expressed mRNAs and lncRNAs from the blue frame in **(B)**.

We then constructed the mRNA-lncRNA co-expression network based on the microarray results. A total 380 lncRNAs and 508 mRNAs containing 3238 relationships were selected to generate a network map (Figure 7A). We identified three core mRNA-lncRNA regulation sub-networks during the process of atherosclerosis exposure to CIH. These sub-networks suggested a complex regulatory relationship between lncRNAs and mRNAs. One lncRNA could regulate multiple mRNAs in different ways, while one mRNA could be regulated by multiple lncRNAs. One sub-network containing 22 lncRNAs and 55 mRNAs is presented in detail in Figure 7B. From the sub-network we found that cytochrome c oxidase subunit 8B (Cox8b), cytochrome c oxidase subunit 7A1 (Cox7a1) and cell death inducing DFFA like effector a (Cidea) were intimately related with lncRNAs Gm12251, Gm5421, Gm13910, and KnowTID_00004506. These mRNAs and lncRNAs were enriched and clustered for further analysis (Figure 7C).

## Discussion

Obstructive sleep apnea causes multiple physiological perturbations, among which CIH, long-term exposure to repeated episodes of hypoxia followed by reoxygenation is considered a prominent feature of OSA pathophysiology (Song et al., 2015). In patients with severe OSA, these repeated episodes of hypoxia and reoxygenation can last for many years with hemoglobin desaturation to a level as low as 50% (Fletcher et al., 1989; Flemons, 2002). In our study, we found that long-term CIH exposure aggravated atherosclerosis in ApoE^–/–^ mice fed a high-fat diet, and CIH followed by normoxia exposure significantly reduced atherosclerosis in ApoE^–/–^ mice compared with mice consistently exposed to long-term CIH exposure (Supplementary Figure S1). These results are consistent with previous studies. Savransky et al. (2007) found that C57BL/6J mice exposed to both high-cholesterol diet (HCD) and CIH developed atherosclerotic lesions in the aortic origin and descending aorta, although mice exposed to CIH alone did not develop atherosclerotic lesions. CIH accelerated atherosclerotic plaque growth in ApoE^–/–^ mice fed a high-cholesterol diet (Jun et al., 2010; Drager et al., 2013). Arnaud et al. (2011) fed ApoE^–/–^ mice a standard-chow diet and exposed them to CIH for 14 days; the authors found that CIH aggravated atherosclerosis more severely in 20-week-old mice compared with 15-week-old mice. Together these studies demonstrated that CIH facilitate atherosclerosis or promote the progression of preexisting atherosclerosis.

Microarray technology enables quick and efficient exploration and identification of expression differences in various biological conditions. In this study, we investigated the mRNAs and lncRNAs in atherosclerotic vascular tissue to explore the molecular mechanisms underlying CIH aggravating atherosclerosis. We found that the number of significantly up-regulated genes was high in mice exposed to CIH for 8 weeks, while the number of significantly up-regulated genes was low in mice exposed to CIH for 12 weeks (Figure 1A). This observation suggests that long-term CIH exposure can have a negative impact on the transcriptome and may lead to a decline in function in the atherosclerotic vascular tissue. To the best of our knowledge, this is the first study to investigate the potential role of differentially expressed mRNAs and lncRNAs in atherosclerotic vascular tissue of CIH exposure mice, and provide important clues to the mechanisms underlying OSA/CIH aggravates atherosclerosis in humans.

In this study, we found that Nr1d1 and Nr1d2 mRNAs were down-regulated in CIH 12W group compared with CIH 8W + Nor 4W group (Table 1). NR1D1 (REV-ERBα) and NR1D2 (REV-ERBβ) are members of the nuclear receptor (NR) superfamily and play important roles in the regulation of circadian rhythm, metabolism and immune function (Kojetin and Burris, 2014). Previous research have shown that hematopoietic knock down of NR1D1 increases atherosclerosis in LDL receptor knockout mice (Ma et al., 2013). SR9009, the synthetic REV-ERB agonist, reduced atherosclerotic plaque size in LDL receptor knockout mice (Sitaula et al., 2015). These researches demonstrated that REV-ERB (including NR1D1 and NR1D2) played an important role in atherosclerosis. Our results show that long-term CIH exposure resulted in decreased Rev-erb mRNA expression (Table 1) and increased atherosclerosis (Supplementary Figure S1), indicating that REV-ERB is likely to play an important role in CIH-aggravated atherosclerosis. Both mRNA Prg4 and lncRNA NONMMUT003096were up-regulated in CIH 8W group compared with Nor 8W (Table 2). Previous research show the Prg4 expression was increased in murine initial atherosclerotic lesions, and deletion of Prg4 in macrophage had effect on both cellular cholesterol metabolism and inflammation *in vitro* (Nahon et al., 2018). Our results demonstrate that Prg4 and NONMMUT003096 had both co-localization and co-expression relationships. The function of NONMMUT003096 is not clear. The previous sequencing data showed that NONMMUT003096 was high expressed in the heart, and expressed in the spleen and lung, but not expressed in the thymus, liver, and hippocampus (Keane et al., 2011). Thus, Prg4 and NONMMUT003096 might have effect on macrophage function in CIH-aggravated atherosclerosis.

Long-term CIH exposure induced up-regulated mRNAs, lncRNA co-localized mRNAs and lncRNA co-expressed mRNAs were all enriched in the pathway of protein processing in endoplasmic reticulum (Figures 4–6). Many of the genes involved in this pathway are ultimately transcribed and translated into heat shock proteins (HSPs) such as HSP90, HSPa8, HSPa5 (Figure 4G). HSPs are evolutionarily conserved and found in most prokaryotes and eukaryotes (Jaattela, 1999). HSPs are induced by physiological and environmental insults such as heat stress, nutritional deficiencies, hypoxia, chemical toxicity, or other types of stress (Chatterjee and Burns, 2017). The main role of HSPs is to act as molecular chaperones that prevent protein misfolding and aggregation by facilitating protein folding and maintaining proper three-dimensional protein structure (Beere, 2004). HSPs not only maintain protein homeostasis, but also maintain cellular homeostasis through their anti-apoptotic effects (Hua et al., 2017). Previous research demonstrated that serum HSP70 (HSPA8) was significantly increased in OSA patients compared with control subjects (Hayashi et al., 2006). Gene expression analysis showed HSP70 was up-regulated in rat gastric epithelial cells under hypoxia-reoxygenation stimulation (Katada et al., 2004). Hypoxia, especially intermittent hypoxia, could induce GRP78 (HSPA5) high expression in aortic arch (Yang et al., 2013). The effects of HSPs on the development of atherosclerosis have been demonstrated in some studies. The inhibitors of HSP90 suppressed the migration of vascular smooth muscle cells and attenuated the formation of atherosclerotic plaques (Kim et al., 2014). HSP70 overexpression increased lipid accumulation in the arteries and promoted the formation of atherosclerotic lesions in ApoE-deficient mice (Zhao et al., 2018). Here we demonstrated that long-term CIH exposure leads to increased expression of HSPs. Because HSPs are essential for the homeostasis of proteins and cells, they are likely to play important roles in CIH-aggravated atherosclerosis.

Brown adipose tissue (BAT) plays an important role in inhibiting atherosclerosis. From the lncRNA-mRNA co-expression sub-network (Figure 7B), we found that Cox8b, Cox7a1 and Cidea were co-expressed with multiple lncRNAs, forming a complex network. Our results demonstrated that CIH leads to a decreased expression of brown adipocyte-selective genes Ucp1 (Table 1), Cox8b, Cox7a1 and Cidea (Figure 7C). CIEDA were significantly down-regulated in atherosclerotic plaques of atherosclerotic patients compared with that in arteries of healthy control (Sulkava et al., 2017). UCP1 in BAT was significantly decreased after exposed to intermittent hypoxia (Fiori et al., 2014). Perivascular adipose tissue (PVAT), which is defined as adipose tissue surrounding the blood vessels, is similar to classical BAT in rodents (Chang et al., 2012). Previous research has demonstrated that impaired PVAT is sufficient to drive increased atherosclerosis (Xiong et al., 2018). BAT activation enhances energy expenditure and decreases plasma triglyceride and cholesterol levels, subsequently protecting against atherosclerosis development (Berbee et al., 2015). Severe brown fat lipoatrophy aggravates the development of atherosclerosis, characterized by increased lipid depots, increases lesion size, and increases the inflammatory response (Gomez-Hernandez et al., 2016). Our results indicated that CIH resulted in decreased browning of PVAT. The decreased browning of PVAT is likely to play an important function in CIH-aggravated atherosclerosis. Our results demonstrated that Cox8b, Cox7a1 and Cidea were intimately related with lncRNAs Gm12251, Gm5421, Gm13910, and KnowTID_00004506 (Figure 7B). The effects of these lncRNAs are not clear. Gm12251 is NADH dehydrogenase (ubiquinone) Fe-S protein 3 pseudogene. Gm5421 is mitochondrial aconitase 2 pseudogene. Gm13910 is hydroxyacyl-CoA dehydrogenase trifunctional multienzyme complex subunit beta pseudogene. These pseudogenes may have effects on regulating parent genes. The previous sequencing data showed that KnowTID_00004506 was not expressed in the heart, spleen, lung, liver, and hippocampus (Keane et al., 2011). As CIH decreased browning of PVAT, these co-expressed lncRNAs (Gm12251, Gm5421, Gm13910, and KnowTID_00004506) may play an important role in CIH aggravating atherosclerosis by regulating expression of Cox8b, Cox7a1, or Cidea. Further studies are needed to validate our findings and to understand the molecular mechanisms of specific lncRNAs implicated in CIH aggravating atherosclerosis.

## Conclusion

Our study provided comprehensive analysis of the mRNA and lncRNA expression profiles in atherosclerotic aorta exposed to CIH. The integrated interpretation of differential mRNA and lncRNA expression reveals that long-term CIH induced the protein processing in endoplasmic reticulum pathway. HSPs and their functions in protein folding may play important roles in CIH aggravating atherosclerosis. We built a molecular interaction network with 380 lncRNAs, 508 mRNAs and 3238 relationships. To the best of our knowledge, this is the first study to investigate the potential role of differentially expressed mRNAs and lncRNAs in atherosclerotic vascular tissue of CIH exposure mice, and provide potential molecular candidates that might be important for future studies on the underlying mechanisms of CIH aggravating atherosclerosis.

## Data Availability Statement

The datasets generated for this study can be found in the NCBI with the GEO accession code GSE145221.

## Ethics Statement

The animal study was reviewed and approved by Institutional Animal Care and Use Committee of Capital Medical University.

## Author Contributions

YQ and YW conceived and designed the experiments. CH and YY performed experiments. XJ and JL performed histopathology analysis. HY analyzed the formality. JZ and CH analyzed the data. JZ wrote the manuscript. All authors reviewed the manuscript.

## Conflict of Interest

The authors declare that the research was conducted in the absence of any commercial or financial relationships that could be construed as a potential conflict of interest.

## Footnotes

1 http://www.geneontology.org

2 http://www.genome.jp/kegg/

## References

[B1] Arnaud C. Poulain L. Levy P. Dematteis M. (2011). Inflammation contributes to the atherogenic role of intermittent hypoxia in apolipoprotein-E knock out mice. *Atherosclerosis* 219 425–431. 10.1016/j.atherosclerosis.2011.07.122 21917260

[B2] Baguet J. P. Hammer L. Levy P. Pierre H. Launois S. Mallion J. M. (2005). The severity of oxygen desaturation is predictive of carotid wall thickening and plaque occurrence. *Chest* 128 3407–3412. 10.1378/chest.128.5.3407 16304292

[B3] Beere H. M. (2004). “The stress of dying”: the role of heat shock proteins in the regulation of apoptosis. *J. Cell Sci.* 117 2641–2651. 10.1242/jcs.01284 15169835

[B4] Berbee J. F. Boon M. R. Khedoe P. P. Bartelt A. Schlein C. Worthmann A. (2015). Brown fat activation reduces hypercholesterolaemia and protects from atherosclerosis development. *Nat. Commun.* 6:6356. 10.1038/ncomms7356 PMC4366535 25754609

[B5] Bradley T. D. Floras J. S. (2009). Obstructive sleep apnoea and its cardiovascular consequences. *Lancet* 373 82–93. 10.1016/s0140-6736(08)61622-0 19101028

[B6] Chang L. Villacorta L. Li R. Hamblin M. Xu W. Dou C. (2012). Loss of perivascular adipose tissue on peroxisome proliferator-activated receptor-gamma deletion in smooth muscle cells impairs intravascular thermoregulation and enhances atherosclerosis. *Circulation* 126 1067–1078. 10.1161/circulationaha.112.104489 22855570 PMC3493564

[B7] Chatterjee S. Burns T. F. (2017). Targeting heat shock proteins in cancer: a promising therapeutic approach. *Int. J. Mol. Sci.* 18:1978 10.3390/ijms18091978 PMC5618627 28914774

[B8] Dempsey J. A. Veasey S. C. Morgan B. J. O’Donnell C. P. (2010). Pathophysiology of sleep apnea. *Physiol. Rev.* 90 47–112. 20086074 10.1152/physrev.00043.2008 PMC3970937

[B9] Drager L. F. Bortolotto L. A. Figueiredo A. C. Krieger E. M. Lorenzi G. F. (2007a). Effects of continuous positive airway pressure on early signs of atherosclerosis in obstructive sleep apnea. *Am. J. Respir. Crit. Care Med.* 176 706–712. 17556718 10.1164/rccm.200703-500OC

[B10] Drager L. F. Bortolotto L. A. Figueiredo A. C. Silva B. C. Krieger E. M. Lorenzi-Filho G. (2007b). Obstructive sleep apnea, hypertension, and their interaction on arterial stiffness and heart remodeling. *Chest* 131 1379–1386. 10.1378/chest.06-2703 17494787

[B11] Drager L. F. Bortolotto L. A. Maki-Nunes C. Trombetta I. C. Alves M. J. Fraga R. F. (2010). The incremental role of obstructive sleep apnoea on markers of atherosclerosis in patients with metabolic syndrome. *Atherosclerosis* 208 490–495. 10.1016/j.atherosclerosis.2009.08.016 19762024

[B12] Drager L. F. Yao Q. Hernandez K. L. Shin M. K. Bevans-Fonti S. Gay J. (2013). Chronic intermittent hypoxia induces atherosclerosis via activation of adipose angiopoietin-like 4. *Am. J. Respir. Crit. Care Med.* 188 240–248. 10.1164/rccm.201209-1688oc 23328524 PMC3778753

[B13] Duran J. Esnaola S. Rubio R. Iztueta A. (2001). Obstructive sleep apnea-hypopnea and related clinical features in a population-based sample of subjects aged 30 to 70 yr. *Am. J. Respir. Crit. Care Med.* 163 685–689. 10.1164/ajrccm.163.3.2005065 11254524

[B14] Fang G. Song D. Ye X. Mao S. Z. Liu G. Liu S. F. (2012). Chronic intermittent hypoxia exposure induces atherosclerosis in ApoE knockout mice: role of NF-kappaB p50. *Am. J. Pathol.* 181 1530–1539. 10.1016/j.ajpath.2012.07.024 22940439

[B15] Fiori C. Z. Martinez D. Baronio D. da Rosa D. P. Kretzmann N. A. Forgiarini L. F. (2014). Downregulation of uncoupling protein-1 mRNA expression and hypoadiponectinemia in a mouse model of sleep apnea. *Sleep Breath.* 18 541–548. 10.1007/s11325-013-0916-2 24337908

[B16] Flemons W. W. (2002). Clinical practice. Obstructive sleep apnea. *N. Engl. J. Med.* 347 498–504. 12181405 10.1056/NEJMcp012849

[B17] Fletcher E. C. Costarangos C. Miller T. (1989). The rate of fall of arterial oxyhemoglobin saturation in obstructive sleep apnea. *Chest* 96 717–722. 10.1378/chest.96.4.717 2791663

[B18] Fruchart J. C. Nierman M. C. Stroes E. S. Kastelein J. J. Duriez P. (2004). New risk factors for atherosclerosis and patient risk assessment. *Circulation* 109 III15–III19. 15198961 10.1161/01.CIR.0000131513.33892.5b

[B19] Gami A. S. Olson E. J. Shen W. K. Wright R. S. Ballman K. V. Hodge D. O. (2013). Obstructive sleep apnea and the risk of sudden cardiac death: a longitudinal study of 10,701 adults. *J. Am. Coll. Cardiol.* 62 610–616. 23770166 10.1016/j.jacc.2013.04.080 PMC3851022

[B20] Gomez-Hernandez A. Beneit N. Escribano O. Diaz-Castroverde S. Garcia-Gomez G. Fernandez S. (2016). Severe brown fat lipoatrophy aggravates atherosclerotic process in male mice. *Endocrinology* 157 3517–3528. 10.1210/en.2016-1148 27414981

[B21] Grammatikakis I. Panda A. C. Abdelmohsen K. Gorospe M. (2014). Long noncoding RNAs(lncRNAs) and the molecular hallmarks of aging. *Aging* 6 992–1009. 25543668 10.18632/aging.100710 PMC4298369

[B22] Guttman M. Rinn J. L. (2012). Modular regulatory principles of large non-coding RNAs. *Nature* 482 339–346. 10.1038/nature10887 22337053 PMC4197003

[B23] Hayashi M. Fujimoto K. Urushibata K. Takamizawa A. Kinoshita O. Kubo K. (2006). Hypoxia-sensitive molecules may modulate the development of atherosclerosis in sleep apnoea syndrome. *Respirology* 11 24–31. 10.1111/j.1440-1843.2006.00780.x 16423198

[B24] Heron M. Hoyert D. L. Murphy S. L. Xu J. Kochanek K. D. Tejada-Vera B. (2009). Deaths: final data for 2006. *Natl. Vital. Stat. Rep.* 57 1–134. 19788058

[B25] Hua C. Ju W. N. Jin H. Sun X. Zhao G. (2017). Molecular chaperones and hypoxic-ischemic encephalopathy. *Neural Regen. Res.* 12 153–160. 28250763 10.4103/1673-5374.199008 PMC5319223

[B26] Hung T. Wang Y. Lin M. F. Koegel A. K. Kotake Y. Grant G. D. (2011). Extensive and coordinated transcription of noncoding RNAs within cell-cycle promoters. *Nat. Genet.* 43 621–629. 10.1038/ng.848 21642992 PMC3652667

[B27] Jaattela M. (1999). Heat shock proteins as cellular lifeguards. *Ann. Med.* 31 261–271. 10.3109/07853899908995889 10480757

[B28] Jian L. Jian D. Chen Q. Zhang L. (2016). Long noncoding RNAs in atherosclerosis. *J. Atheroscler. Thromb.* 23 376–384. 26699715 10.5551/jat.33167

[B29] Jun J. Reinke C. Bedja D. Berkowitz D. Bevans-Fonti S. Li J. (2010). Effect of intermittent hypoxia on atherosclerosis in apolipoprotein E-deficient mice. *Atherosclerosis* 209 381–386. 10.1016/j.atherosclerosis.2009.10.017 19897196 PMC2846209

[B30] Katada K. Naito Y. Shimozawa M. Mizushima K. Kuroda M. Takagi T. (2004). Gene expression analysis following hypoxia-reoxygenation in rat gastric epithelial cells using a high-density oligonucleotide array. *Redox Rep.* 9 337–342. 10.1179/135100004225006849 15720829

[B31] Keane T. M. Goodstadt L. Danecek P. White M. A. Wong K. Yalcin B. (2011). Mouse genomic variation and its effect on phenotypes and gene regulation. *Nature* 477 289–294. 21921910 10.1038/nature10413 PMC3276836

[B32] Khattak H. K. Hayat F. Pamboukian S. V. Hahn H. S. Schwartz B. P. Stein P. K. (2018). Obstructive sleep apnea in heart failure: review of prevalence, treatment with continuous positive airway pressure, and prognosis. *Tex Heart Inst. J.* 45 151–161. 10.14503/thij-15-5678 30072851 PMC6059510

[B33] Kim J. Jang S. W. Park E. Oh M. Park S. Ko J. (2014). The role of heat shock protein 90 in migration and proliferation of vascular smooth muscle cells in the development of atherosclerosis. *J. Mol. Cell Cardiol.* 72 157–167. 10.1016/j.yjmcc.2014.03.008 24650873

[B34] Kohler M. Stradling J. R. (2010). Mechanisms of vascular damage in obstructive sleep apnea. *Nat. Rev. Cardiol.* 7 677–685. 10.1038/nrcardio.2010.145 21079639

[B35] Kojetin D. J. Burris T. P. (2014). REV-ERB and ROR nuclear receptors as drug targets. *Nat. Rev. Drug Discov.* 13 197–216. 10.1038/nrd4100 24577401 PMC4865262

[B36] Lavie P. Herer P. Hoffstein V. (2000). Obstructive sleep apnoea syndrome as a risk factor for hypertension: population study. *BMJ* 320 479–482. 10.1136/bmj.320.7233.479 10678860 PMC27290

[B37] Levy P. Kohler M. McNicholas W. T. Barbe F. McEvoy R. D. Somers V. K. (2015). Obstructive sleep apnoea syndrome. *Nat. Rev. Dis. Primers* 1:15015. 10.1038/nrdp.2015.15 27188535

[B38] Liu Y. Zheng L. Wang Q. Hu Y. W. (2017). Emerging roles and mechanisms of long noncoding RNAs in atherosclerosis. *Int. J. Cardiol.* 228 570–582. 10.1016/j.ijcard.2016.11.182 27875736

[B39] Ma H. Zhong W. Jiang Y. Fontaine C. Li S. Fu J. (2013). Increased atherosclerotic lesions in LDL receptor deficient mice with hematopoietic nuclear receptor Rev-erbalpha knock- down. *J. Am. Heart Assoc.* 2:e000235. 10.1161/JAHA.113.000235 PMC3828791 23963755

[B40] Ma L. Zhang J. Liu Y. (2016). Roles and mechanisms of obstructive sleep apnea-hypopnea syndrome and chronic intermittent hypoxia in atherosclerosis: evidence and prospective. *Oxid. Med. Cell Longev.* 2016:82 15082. 10.1155/2016/8215082 PMC4884866 27293515

[B41] Nahon J. E. Hoekstra M. Havik S. R. Van Santbrink P. J. Dallinga-Thie G. M. Kuivenhoven J. A. (2018). Proteoglycan 4 regulates macrophage function without altering atherosclerotic lesion formation in a murine bone marrow-specific deletion model. *Atherosclerosis* 274 120–127. 10.1016/j.atherosclerosis.2018.05.008 29772480

[B42] Neubauer J. A. (2001). Invited review: physiological and pathophysiological responses to intermittent hypoxia. *J. Appl. Physiol.* 90 1593–1599. 10.1152/jappl.2001.90.4.1593 11247965

[B43] Pan Z. Li L. Fang Q. Zhang Y. Hu X. Qian Y. (2018). Analysis of dynamic molecular networks for pancreatic ductal adenocarcinoma progression. *Cancer Cell Int.* 18:214. 10.1186/s12935-018-0718-5 PMC6303882 30598639

[B44] Peker Y. Hedner J. Norum J. Kraiczi H. Carlson J. (2002). Increased incidence of cardiovascular disease in middle-aged men with obstructive sleep apnea: a 7-year follow-up. *Am. J. Respir. Crit. Care Med.* 166 159–165. 10.1164/rccm.2105124 12119227

[B45] Rossi V. A. Stradling J. R. Kohler M. (2013). Effects of obstructive sleep apnoea on heart rhythm. *Eur. Respir. J.* 41 1439–1451. 10.1183/09031936.00128412 23258782

[B46] Savransky V. Nanayakkara A. Li J. Bevans S. Smith P. L. Rodriguez A. (2007). Chronic intermittent hypoxia induces atherosclerosis. *Am. J. Respir. Crit. Care Med.* 175 1290–1297. 17332479 10.1164/rccm.200612-1771OC PMC2176090

[B47] Shah N. A. Yaggi H. K. Concato J. Mohsenin V. (2010). Obstructive sleep apnea as a risk factor for coronary events or cardiovascular death. *Sleep Breath.* 14 131–136. 10.1007/s11325-009-0298-7 19777281

[B48] Sitaula S. Billon C. Kamenecka T. M. Solt L. A. Burris T. P. (2015). Suppression of atherosclerosis by synthetic REV-ERB agonist. *Biochem. Biophys. Res. Commun.* 460 566–571. 10.1016/j.bbrc.2015.03.070 25800870 PMC4855281

[B49] Song D. Fang G. Greenberg H. Liu S. F. (2015). Chronic intermittent hypoxia exposure-induced atherosclerosis: a brief review. *Immunol. Res.* 63 121–130. 10.1007/s12026-015-8703-8 26407987

[B50] Song L. Langfelder P. Horvath S. (2012). Comparison of co-expression measures: mutual information, correlation, and model based indices. *BMC Bioinformatics* 13:328 10.1186/1471-2105-13-328 PMC3586947 23217028

[B51] Stuart J. M. Segal E. Koller D. Kim S. K. (2003). A gene-coexpression network for global discovery of conserved genetic modules. *Science* 302 249–255. 10.1126/science.1087447 12934013

[B52] Sulkava M. Raitoharju E. Levula M. Seppala I. Lyytikainen L. P. Mennander A. (2017). Differentially expressed genes and canonical pathway expression in human atherosclerotic plaques - Tampere Vascular Study. *Sci. Rep.* 7:41483. 10.1038/srep41483 PMC5270243 28128285

[B53] Tabas I. Garcia-Cardena G. Owens G. K. (2015). Recent insights into the cellular biology of atherosclerosis. *J. Cell Biol.* 209 13–22. 10.1083/jcb.201412052 25869663 PMC4395483

[B54] Xiong W. Zhao X. Villacorta L. Rom O. Garcia-Barrio M. T. Guo Y. (2018). Brown adipocyte-specific PPARgamma (peroxisome proliferator-activated receptor gamma) deletion impairs perivascular adipose tissue development and enhances atherosclerosis in mice. *Arterioscler. Thromb. Vasc. Biol.* 38 1738–1747. 10.1161/atvbaha.118.311367 29954752 PMC6202167

[B55] Yaggi H. K. Concato J. Kernan W. N. Lichtman J. H. Brass L. M. Mohsenin V. (2005). Obstructive sleep apnea as a risk factor for stroke and death. *N. Engl. J. Med.* 353 2034–2041. 16282178 10.1056/NEJMoa043104

[B56] Yang Y. Y. Shang J. Liu H. G. (2013). Role of endoplasmic reticular stress in aortic endothelial apoptosis induced by intermittent/persistent hypoxia. *Chin. Med. J.* 126 4517–4523. 24286417

[B57] Young T. Peppard P. E. Gottlieb D. J. (2002). Epidemiology of obstructive sleep apnea: a population health perspective. *Am. J. Respir. Crit. Care Med.* 165 1217–1239. 11991871 10.1164/rccm.2109080

[B58] Zhao Z. W. Zhang M. Chen L. Y. Gong D. Xia X. D. Yu X. H. (2018). Heat shock protein 70 accelerates atherosclerosis by downregulating the expression of ABCA1 and ABCG1 through the JNK/Elk-1 pathway. *Biochim. Biophys. Acta Mol. Cell. Biol. Lipids* 1863 806–822. 10.1016/j.bbalip.2018.04.011 29678642

[B59] Zhou T. Ding J. W. Wang X. A. Zheng X. X. (2016). Long noncoding RNAs and atherosclerosis. *Atherosclerosis* 248 51–61. 26987066 10.1016/j.atherosclerosis.2016.02.025

