# Supplementary material for: Potential Role of mRNAs and LncRNAs in Chronic Intermittent Hypoxia Exposure-Aggravated Atherosclerosis
Source: Front Genet. 2020 Apr 9;11:290. doi: 10.3389/fgene.2020.00290 (PMC7160761; doi:10.3389/fgene.2020.00290)
Supplement: Supplementary file 1 [file Image_1.PDF]

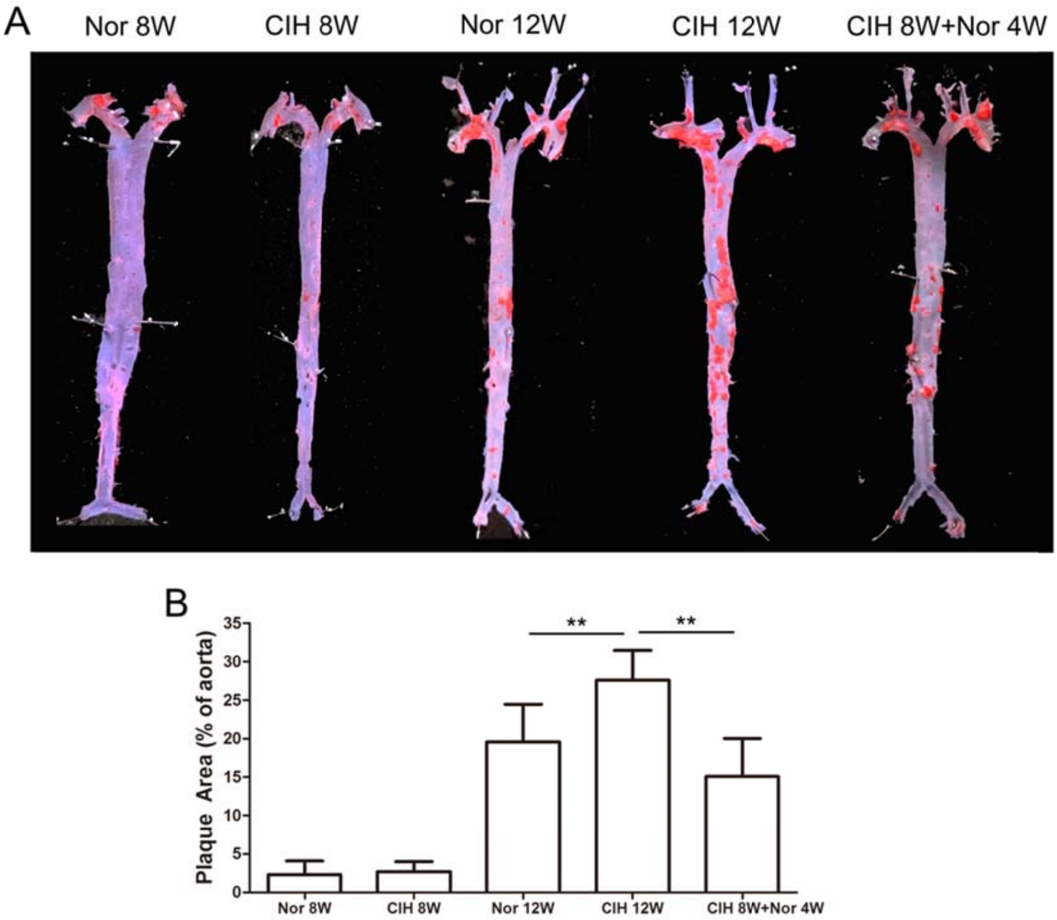

Supplementary Figure 1. CIH exposure aggravates atherosclerosis in ApoE-deficient mice. Plaque formation in mice fed a high-fat diet for 8 or 12 weeks was evaluated by Oil Red O staining. (A) Representative photographs are shown. (B) The plaque area was calculated as a percent of the aorta staining positive for Oil Red O. Nor 8W, ApoE-deficient mice in normoxia for 8 weeks; CIH 8W, ApoE-deficient mice in CIH for 8 weeks; Nor 12W, ApoE-deficient mice in normoxia for 12 weeks; CIH 12W, ApoE-deficient mice in CIH for 12 weeks; CIH 8W+Nor 4W, ApoE-deficient mice in CIH for 8 weeks followed by normoxia for 4 weeks. n=6 in each group. ***p*<0.01.
